# Publisher Correction: Breaking the speed limit with multimode fast scanning of DNA by Endonuclease V

**DOI:** 10.1038/s41467-019-10070-x

**Published:** 2019-04-25

**Authors:** Arash Ahmadi, Ida Rosnes, Pernille Blicher, Robin Diekmann, Mark Schüttpelz, Kyrre Glette, Jim Tørresen, Magnar Bjørås, Bjørn Dalhus, Alexander D. Rowe

**Affiliations:** 1 Department of Medical Biochemistry, Institute for Clinical Medicine, University of Oslo, NO-0372 Oslo, Norway; 2 Department of Microbiology, Oslo University Hospital HF, Rikshospitalet and University of Oslo, PO Box 4950 Nydalen, NO-0424 Oslo, Norway; 3 Biomolecular Photonics, Department of Physics, University of Bielefeld, Universitätsstraße 25, DE-33615 Bielefeld, Germany; 4 European Molecular Biology Laboratory (EMBL), Cell Biology and Biophysics Unit, Meyerhofstraße 1, DE-69117 Heidelberg, Germany; 5 Department of Informatics, University of Oslo, PO Box 1080 Blindern, NO-0316 Oslo, Norway; 6 Department of Clinical and Molecular Medicine, Faculty of Medicine and Health Sciences, Norwegian University of Science and Technology (NTNU), PO Box 8905, NO-7491 Trondheim, Norway; 7 Department of Newborn Screening, Division of Child and Adolescent Medicine, Oslo University Hospital, PO Box 4950 Nydalen, NO-0424 Oslo, Norway

**Keywords:** DNA damage and repair, Single-molecule biophysics

Correction to: *Nature Communications* 10.1038/s41467-018-07797-4, published online 19 December 2018.

The original version of this Article was updated shortly after publication to add a link to the Peer Review file, which was inadvertently omitted. The Peer Review file is available to download as a Supplementary File from the HTML version of the Article.

