# Supplementary material for: Breaking the speed limit with multimode fast scanning of DNA by Endonuclease V
Source: Nat Commun. 2018 Dec 19;9:5381. doi: 10.1038/s41467-018-07797-4 (PMC6300609; doi:10.1038/s41467-018-07797-4)
Supplement: Supplementary file 1 — Supplementary Information [file 41467_2018_7797_MOESM1_ESM.pdf]

Supplementary Information

Breaking the speed limit with multimode fast scanning of DNA by Endonuclease V

Arash Ahmadi et al.

Supplementary Note 1 – diffusion rate calculations

According to the Einstein-Smolluchowski relation, the diffusion constant of a particle in a fluid can be calculated from the hydrodynamic characteristics of the particle and its surrounding medium as[1]

$$D = \frac{k_B T}{f} \qquad (1)$$

where k_B is the Boltzmann constant, T is absolute temperature and f is the frictional drag coefficient. In a study in 1979, Schurr[2] assumed that proteins envelop the DNA, such that their center of mass is aligned with the axis of the DNA, and he calculated a frictional coefficient for rotational-coupled sliding of protein along the DNA as

$$f = 6\pi\eta R + (\frac{2\pi}{10BP})^2 8\pi\eta R^3 \qquad (2)$$

where BP stands for distance between base pairs along the axis of DNA, R is the radius of the protein, and η is the viscosity of the surrounding fluid. The first term in this equation is the translational contribution of frictional coefficient, determined from the net displacement along the DNA axis. The second term represents the rotational contribution of the frictional coefficient due to rotation of the protein around the axis of the DNA. To consider cases in which the center of mass of the protein and the axis of DNA are not aligned a more recent study[3] updated Schurr's calculation as

$$f = 6\pi\eta R + (\frac{2\pi}{10BP})^2 [8\pi\eta R^3 + 6\pi\eta R(R_{oc})^2] \qquad (3)$$

where R_{oc} is the offset between the center of mass of the protein and the axis of the DNA. The third term in this equation is the translational friction due to curvilinear motion along the helix. By combining equation (3) and equation (1), the theoretical diffusion rate for helical sliding along DNA can be calculated. It should be noted that this calculation is based on a purely hydrodynamic model for diffusion and the friction between the DNA and protein is not considered. Therefore, this value represents the upper limit of diffusion for helical sliding. For hOGG1, the values of $R = 3.2\ nm$ and $R_{oc} = 2.5\ nm$ have been previously published. From comparison of the two proteins' masses and direct measurement using published crystal structures, we calculated the values for EndoV as $R = 2.9\ nm$ and $R_{oc} = 2.0\ nm$, respectively. From these values and the above mentioned equations, for hOGG1 and EndoV, we calculated the upper limit of diffusion for helical sliding to be 0.89 $\mu m^2 s^{-1}$ and 1.3$\mu m^2 s^{-1}$, respectively.

The experimentally determined 1D diffusion coefficient can be calculated from trajectories of proteins moving along DNA. The mean square displacement (MSD) of proteins $< x^2 >$ in a one-dimensional Brownian motion is proportional to the diffusion constant D and time t[1]

$$< x^2 > = 2Dt \tag{4}$$

Thus, the experimental diffusion constant can be calculated as $D = < x^2 > /2t$. This formula is used for calculation of the instantaneous diffusion rate using moving windows with limited time frames.

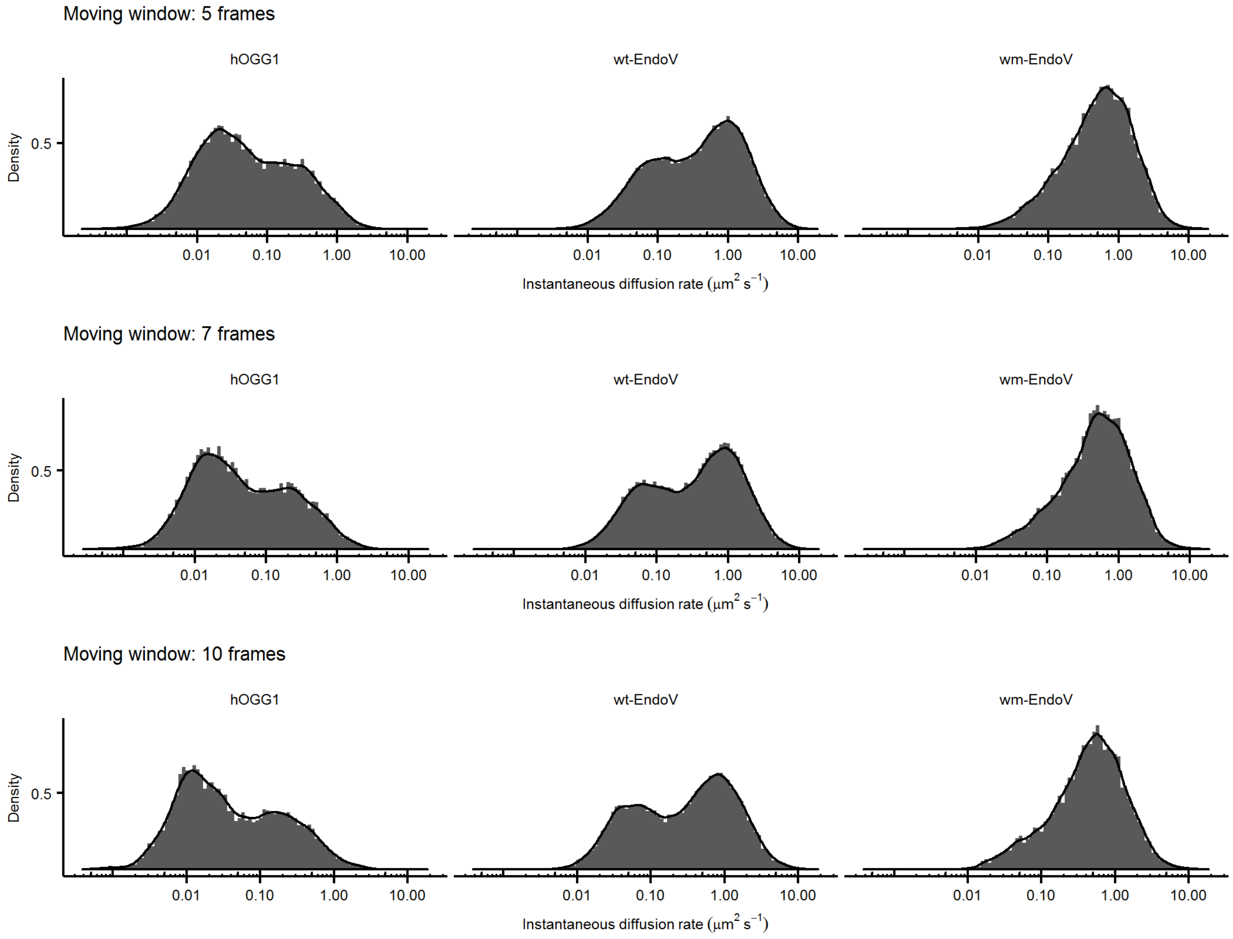

Supplementary Figure 1 | Instantaneous diffusion rate plots. The density distribution of the instantaneous diffusion rates were calculated using moving windows with widths of 5, 7 and 10 frames. The modalities of the distributions are consistent and independent of the chosen window.

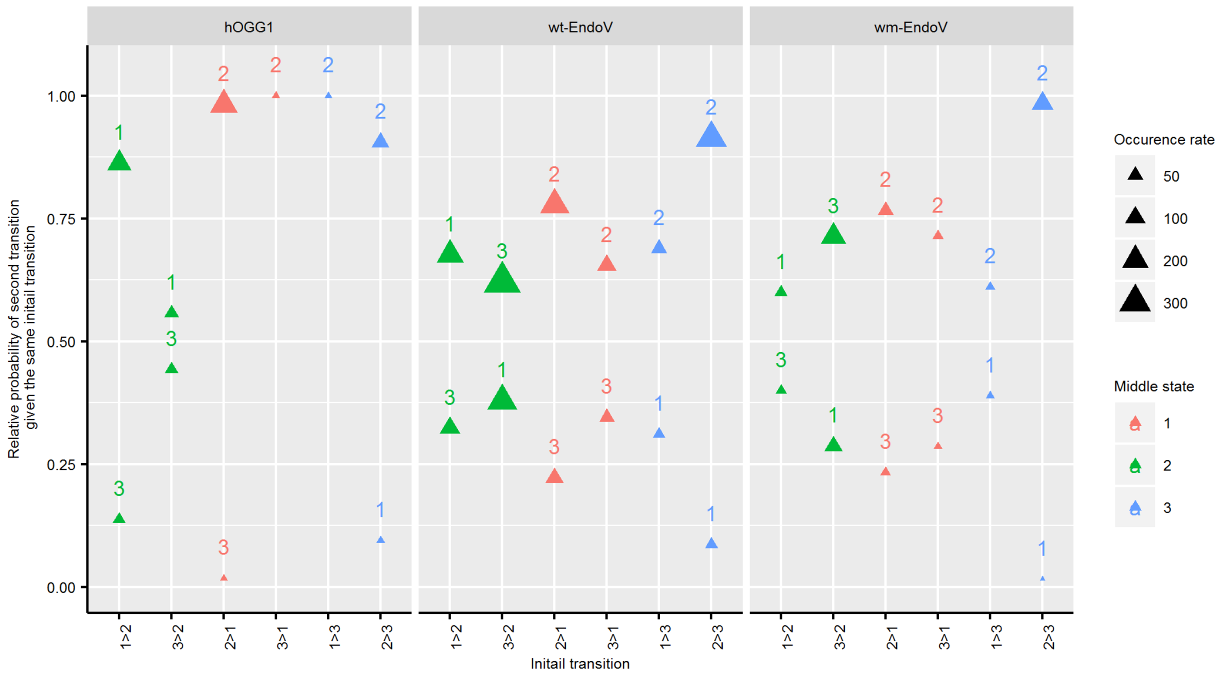

Supplementary Figure 2 | The effect of memory in transitions. The effects of an initial transition on the next transition are shown for series of 2 consecutive transitions. The x-axis shows the initial transitions and the y-axis is the relative probability of the third scanning mode following the second transition.

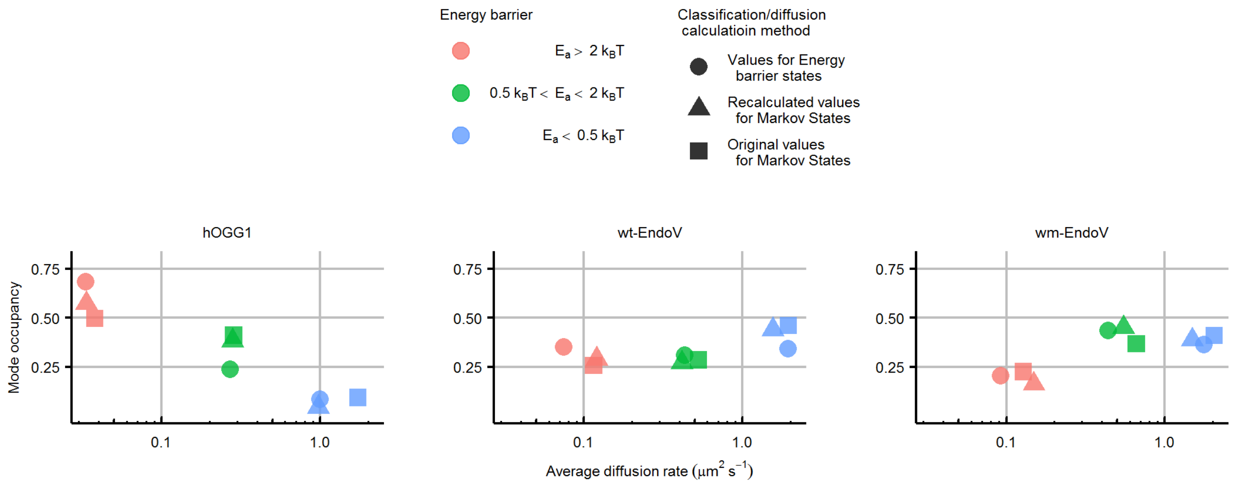

Supplementary Figure 3 | Diffusion mode and occupancy of states. The values of the occupancy and the average diffusion rate of the states are compared for the energy barrier-based and hidden Markov model-based classifications. Original values for the Markov states are the output of the vbSPT software, and the recalculated values are obtained after the frame correction is performed.

Supplementary References

1. Berg, H. C. *Random Walks in Biology*. (Prineton University press, 1993).

2. Schurr, J. M. The one-dimensional diffusion coefficient of proteins absorbed on DNA. Hydrodynamic considerations. *Biophys. Chem.* **9,** 413–414 (1975).

3. Bagchi, B., Blainey, P. C. & Sunney Xie, X. Diffusion constant of a nonspecifically bound protein undergoing curvilinear motion along DNA. *J. Phys. Chem. B* **112,** 6282–6284 (2008).

.
