# Supplementary material for: Breaking the speed limit with multimode fast scanning of DNA by Endonuclease V
Source: Nat Commun. 2018 Dec 19;9:5381. doi: 10.1038/s41467-018-07797-4 (PMC6300609; doi:10.1038/s41467-018-07797-4)
Supplement: Supplementary file 2 — Description of Additional Supplementary Information [file 41467_2018_7797_MOESM2_ESM.docx]

Supplementary Movie legends

Supplementary Movie 1: Representative trajectories of DNA scanning by hOGG1, wt-EndoV and wm-EndoV taken from different data sets and concatenated.

Supplementary Movie 2: Representative trajectories of DNA scanning by wt-EndoV attributed to different diffusion modes.
