# Supplementary material for: Breaking the speed limit with multimode fast scanning of DNA by Endonuclease V
Source: Nat Commun. 2018 Dec 19;9:5381. doi: 10.1038/s41467-018-07797-4 (PMC6300609; doi:10.1038/s41467-018-07797-4)
Supplement: Supplementary file 5 — Reporting Summary [file 41467_2018_7797_MOESM5_ESM.pdf]

nature research

Corresponding author(s): Bjørn Dalhus & Alexander D Rowe

Reporting Summary

Nature Research wishes to improve the reproducibility of the work that we publish. This form provides structure for consistency and transparency in reporting. For further information on Nature Research policies, see Authors & Referees and the Editorial Policy Checklist.

Statistical parameters

When statistical analyses are reported, confirm that the following items are present in the relevant location (e.g. figure legend, table legend, main text, or Methods section).

n/a	Confirmed	
☐	☒	The exact sample size (n) for each experimental group/condition, given as a discrete number and unit of measurement
☐	☒	An indication of whether measurements were taken from distinct samples or whether the same sample was measured repeatedly
☐	☒	The statistical test(s) used AND whether they are one- or two-sided<br>*Only common tests should be described solely by name; describe more complex techniques in the Methods section.*
☐	☒	A description of all covariates tested
☐	☒	A description of any assumptions or corrections, such as tests of normality and adjustment for multiple comparisons
☐	☒	A full description of the statistics including central tendency (e.g. means) or other basic estimates (e.g. regression coefficient) AND variation (e.g. standard deviation) or associated estimates of uncertainty (e.g. confidence intervals)
☐	☒	For null hypothesis testing, the test statistic (e.g. F, t, r) with confidence intervals, effect sizes, degrees of freedom and P value noted<br>*Give P values as exact values whenever suitable.*
☐	☒	For Bayesian analysis, information on the choice of priors and Markov chain Monte Carlo settings
☐	☒	For hierarchical and complex designs, identification of the appropriate level for tests and full reporting of outcomes
☐	☒	Estimates of effect sizes (e.g. Cohen's d, Pearson's r), indicating how they were calculated
☐	☒	Clearly defined error bars<br>*State explicitly what error bars represent (e.g. SD, SE, CI)*

Our web collection on statistics for biologists may be useful.

Software and code

Policy information about availability of computer code

Data collection	ThunderSTORM plugin in FIJI (version 1.51) (Ovesný, M., Křížek, P., Borkovec, J., Švindrych, Z. & Hagen, G. M. ThunderSTORM: A comprehensive ImageJ plug-in for PALM and STORM data analysis and super-resolution imaging. Bioinformatics 30, 2389–2390 (2014) & Schindelin, J. et al. Fiji: an open-source platform for biological-image analysis. Nat. Methods 9, 609 676–682 (2012))<br>Micro-manager (version 1.4.22)
Data analysis	Custom code in R (version 3.5.1) (All source code used in the analysis pipeline are provided with the paper as supplementary information).<br>vbSPT (Persson, F., Lindén, M., Unoson, C. & Elf, J. Extracting intracellular diffusive states and transition rates from single-molecule tracking data. Nat. Methods 10, 265–9 (2013))

For manuscripts utilizing custom algorithms or software that are central to the research but not yet described in published literature, software must be made available to editors/reviewers upon request. We strongly encourage code deposition in a community repository (e.g. GitHub). See the Nature Research guidelines for submitting code & software for further information.

Data

Policy information about availability of data

All manuscripts must include a data availability statement. This statement should provide the following information, where applicable:

- Accession codes, unique identifiers, or web links for publicly available datasets
- A list of figures that have associated raw data
- A description of any restrictions on data availability

The raw image data captured during this study are available from the corresponding authors upon request.

Field-specific reporting

Please select the best fit for your research. If you are not sure, read the appropriate sections before making your selection.

☒ Life sciences ☐ Behavioural & social sciences ☐ Ecological, evolutionary & environmental sciences

For a reference copy of the document with all sections, see nature.com/authors/policies/ReportingSummary-flat.pdf

Life sciences study design

All studies must disclose on these points even when the disclosure is negative.

Sample size	No sample-size calculations were performed. The number of samples studied were determined by the number of observed single-molecule interactions in the combined data set for each protein.
Data exclusions	No data were excluded from the study, except for trajectories where no signal could be detected as described in the methods.
Replication	Each single-molecule protein-DNA interaction was studied using several samples of DNA and proteins, and the analysis of the molecular behavior was performed on the combined data sets encompassing all data collected.
Randomization	Randomization of samples is not relevant in this study as we want to analyse the combined behavior of each molecule in contact with DNA.
Blinding	Blinding is not relevant in this study as we want to analyse the combined behavior of each molecule in contact with DNA

Reporting for specific materials, systems and methods

Materials & experimental systems

n/a	Involved in the study	
☐	☒	Unique biological materials
☐	☒	Antibodies
☒	☐	Eukaryotic cell lines
☒	☐	Palaeontology
☒	☐	Animals and other organisms
☒	☐	Human research participants

Methods

n/a	Involved in the study	
☒	☐	ChIP-seq
☒	☐	Flow cytometry
☒	☐	MRI-based neuroimaging

Unique biological materials

Policy information about availability of materials

Obtaining unique materials	All material is available from commercial sources (DNA template, primes, dye, linkers, microsphere beads, antibodies, chemicals for immobilization and surface functionalization) or from the authors on request (plasmids for protein expression).

Antibodies

Antibodies used	Streptavidine (85878-5MG, Sigma-Aldrich) and Anti-dogoxygenine (11214667001, Fab fragments from sheep, Roche)
Validation	Anti-dig: The polyclonal antibody from sheep is specific to digoxigenin and digoxin and shows no cross-reactivity with other steroids, such as human estrogens and androgens.
